# Systematic review of registered trials of Hydroxychloroquine prophylaxis for COVID-19 health-care workers at the first third of 2020

**DOI:** 10.1016/j.onehlt.2020.100141

**Published:** 2020-05-19

**Authors:** Anne-Lise Bienvenu, Aileen M. Marty, Malcolm K. Jones, Stephane Picot

**Affiliations:** a Groupement Hospitalier Nord, Service Pharmacie, Hospices Civils de Lyon, Lyon, France; b Univ Lyon, Malaria Research Unit, ICBMS, UMR 5246 CNRS INSA CPE, F-69100, Lyon, France; c Translational Medicine; HWCOM, FIU Health Travel Medicine Program and Vaccine Clinic Commander, Emergency Response Team Development, Miami, Florida; d School of Veterinary Science, The University of Queensland, Brisbane, Qld, Australia; e Institute of Parasitology and Medical Mycology, Croix-Rousse Hospital, Hospices Civils de Lyon, 69004 Lyon, France

**Keywords:** Hydroxychloroquine, COVID-19, SARS-CoV2, Pre-exposure prophylaxis, Post-exposure prophylaxis, clinicaltrials.gov

## Abstract

In the absence of a vaccine the medical and scientific community is looking intensely at utilizing a pre or post exposure drug that could decrease viremia. The search for a medication that could reduce risk of serious disease, and ideally of any manifestation of disease from SARS-CoV2, and of asymptomatic shedding of SARS-CoV2 is of urgent interest. Repurposing existing pharmaceuticals is among the approaches to achieve these ends. We performed a systematic review of all interventional studies registered in ClinicalTrials.gov with a focus on one repurposed drug, Hydroxychloroquine (HCQ). The detailed analysis of these studies, some of them already recruiting, provide an overall picture of HCQ use as a COVID-19 prophylaxis around the world. Among the included studies, all but three were randomized and parallel and most of them (74%, 23/31) were double-blinded to quadruple-blinded studies. We found a great diversity in dosing and nearly all the possible scientifically reasonable regimens are under evaluation. This diversity offers benefits as well as challenges. Importantly, the final analysis of these trials should be done through an extensive reading of the results in regard to the clinical design, it will be crucial to carefully read and evaluate the results of each study in regards to the clinical design rather than quickly glancing a 140 characters-based social media message announcing the failure or success of a drug against a disease.

## 1 Introduction

In the absence of a vaccine the medical and scientific community is looking intensely at utilizing a pre or post exposure drug that could decrease viremia. The search for a medication that could reduce risk of serious disease, and ideally of any disease and of asymptomatic shedding of SARS-CoV2 is of urgent interest, particularly to decrease the risk to health care workers, first responders, and others with high risk of exposure to patients with COVID19. The prospect of protecting health-care workers against COVID-19 based on repurposing of existing pharmaceuticals is among one of the recent scientific debates [1,2]. The value of hydroxychloroquine (HCQ) as a prophylactic needs careful documented, empirical research in this context [3]. In order to obtain strong clinical evidence, a large number of scientists and teams have launched prospective studies that were registered on ClinicalTrials.gov over a short period of time. The detailed analysis of these studies, some of them already recruiting, will give an overall picture of HCQ use as a COVID-19 prophylaxis around the world. This will help to identify the gaps to be fulfilled with the idea of getting definitive evidence on the positioning of HCQ for COVID-19 prophylaxis in exposed health-care workers.

## 2 Material and methods

We performed a systematic review of all interventional studies registered in ClinicalTrials.gov on the 27th of April under the disease “COVID” and “hydroxychloroquine prophylaxis” as other terms [4]. No other filter was used. Studies using hydroxychloroquine (HCQ) as treatment, studies that did not record details about HCQ regimen, as well as those using HCQ in combination with other drugs, were not included. ClinicalTrials.gov is a Web-based resource maintained by the National Library of Medicine that provides patients, their family members, health care professionals, researchers, and the public with access to information on clinical studies. Information on ClinicalTrials.gov is provided and updated by the sponsor or principal investigator of the clinical study.

Two independent authors (ALB, SP) performed the screening of the study record detail to assess eligibility. Data were extracted by Information collected included ClinicalTrials.gov identifier, official title, recruitment status, starting and completion dates, estimated enrolment, allocation, location, intervention model, masking, and HCQ regimen. To ensure reproducibility and completeness of data extraction, an Excel spreadsheet (Microsoft Corp., Redmond, WA, USA) compiling all variables to be extracted was used. Disagreements over eligibility or data extraction were resolved by discussion. Data were centrally checked by an independent operator for completeness, plausibility, and integrity before synthesis.

## 3 Results

**Fig. 1:**
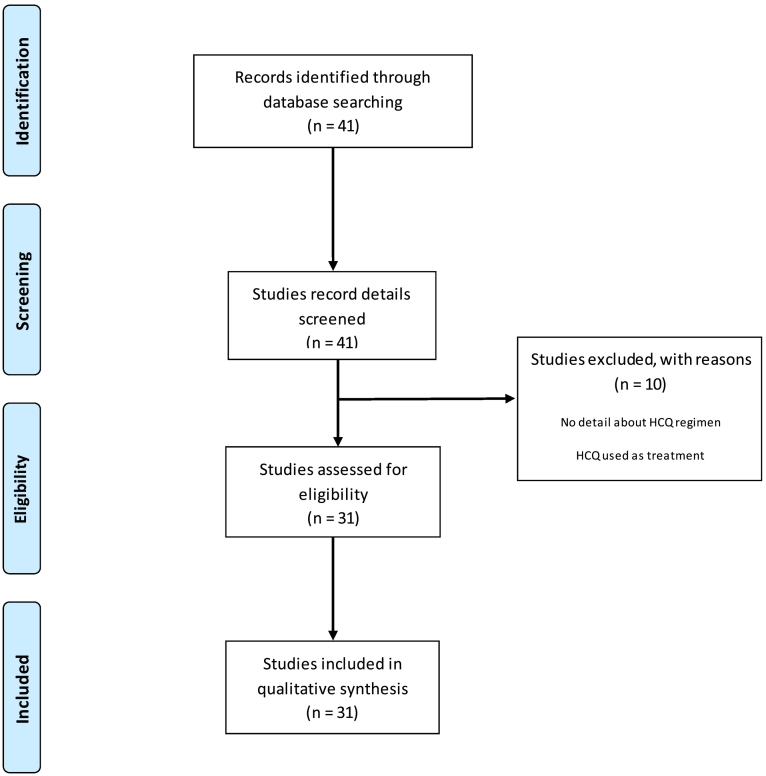
PRISMA 2009 Flow Diagram.

All interventional clinical trials that studied the use of HCQ for COVID-19 prophylaxis were included in the qualitative analysis. Forty-one (*n* = 41) studies were identified through ClinicalTrials.gov on the 27th of April (Fig. 1). After screening for eligibility record details of the selected studies, 31 studies were included in the qualitative analysis. Ten studies were not included: reasons for exclusion included the absence of details about HCQ regimen (*n* = 1), the use of HCQ as indication other than prophylaxis (*n* = 3), and the combination of HCQ to other drugs or vitamins (*n* = 6). The qualitative analysis focussed on HCQ drug regimens of the 31 included studies as recorded in ClinicalTrials.gov from the 17th of March to the 24th of April. (See Table 1.)

**Table 1:** Description of interventional studies registered in ClinicalTrials.gov on the 27th of April under the disease “COVID” and “hydroxychloroquine prophylaxis”.

**NCT**	**Title**	**Starting Date**	**Completion date**	**Recruting**	**Location**	**Randomization**	**Masking**	**Placebo**	**Nb patients**	**Nb in HCQ arm**	**Dose D1** **mg**	**Dose per day mg**	**Nb days**	**Dose per week**	**Nb of weeks**
4,308,668	Post-exposure Prophylaxis or Preemptive Therapy for SARS-Coronavirus-2: A Pragmatic Randomized Clinical Trial	March 17, 2020	April 21, 2020	Yes	Minneapolis, Minnesota, USA	Yes	Quadruple	Yes	3000	1500	**1400**	**600**	**5**	mg	**–**
4,334,148	Healthcare Worker Exposure Response and Outcomes of Hydroxychloroquine Trial	April 2020	July 2020	No	Duke University Durham, UK	Yes	Triple	Yes	15,000	7500	**1200**	**400**	**30**	mg	**–**
4,342,221	Randomized Controlled Trial of Hydroxychloroquine Versus Placebo for the Treatment of Adult Patients With Acute Coronavirus Disease 2019 - COVID-19	March 29, 2020	March 2021	Yes	University Hospital Tuebingen, Germany	Yes	Quadruple	Yes	220	110	**800**	**600**	**7**	mg	**–**
4,329,923	Prevention And Treatment of COVID-19 With Hydroxychloroquine	April 2020	April 2021	Yes	University of Pennsylvania, USA	Yes	Triple	Yes	400	200	**600**	**600**	**60**	mg	**–**
4,353,037	PATCH 2 & 3: Prevention and Treatment of COVID-19 With Hydroxychloroquine) An Open Label Multi-arm Randomized Trial of Hydroxychloroquine in the Prevention and Treatment of COVID-19	April 7, 2020	April 2021	Yes	ProHealth New York, USA	Yes	Double	Yes	850	210	**600**	**600**	**60**	mg	**–**
4,352,933	ChemoPROphyLaxIs For covId-19 Infectious Disease (the PROLIFIC Trial)	April 2020	October 31, 2020	No	Cambridge University Hospitals NHS Foundation Trust, UK	Yes	Quadruple	Yes	1000	330	**800 D1 & D2**	**200**	**90**	**or 400/wk after loading**	**12**
4,330,144	Hydroxychloroquine as Post Exposure Prophylaxis for SARS-CoV-2	01/04/2020	30/03/2021	No	Gangnan severance hospital, Seoul, South Korea	Yes	Single	No	2486	1243	**800**	**400**	**5**	mg	**–**
4,318,444	Hydroxychloroquine Post Exposure Prophylaxis (PEP) for Household Contacts of COVID-19 Patients: A NYC Community-Based Randomized Clinical Trial	March 2020	March 2021	No	Columbia University Irving Medical Center New York, USA	Yes	Quadruple	Yes	1600	800	**800**	**400**	**5**	mg	**–**
4,342,156	Safety And Efficacy Of Hydroxychloroquine As COVID-19 Prophylaxis For At-Risk Population	April 2020	August 2020	No	Tan Tock Seng Hospital Singapore	Yes	None	No	1200	600	**800**	**400**	**5**	mg	**–**
4,304,053	Treatment of Non-severe Confirmed Cases of COVID-19 and Chemoprophylaxis of Their Contacts as Prevention Strategy: a Cluster Randomized Clinical Trial (PEP CoV-2 Study)	March 18, 2020	June 15, 2020	Yes	Departament de Salut, Barcelona, Spain	Yes	None	No	3040	1520	**800**	**400**	**5**	mg	**–**
4,329,611	A Randomized, Double-blind, Placebo-controlled Trial to Assess the Efficacy and Safety of Oral Hydroxychloroquine for the Treatment of SARS-CoV-2 Positive Patients for the Prevention of Severe COVID-19 Disease.	April 13	August 31, 2020	Yes	University of Calgary/Foothills Medical Centre, Calgary, Alberta, Canada	Yes	Triple	Yes	1660	550	**800**	**400**	**5**	mg	**–**
4,351,191	Use and Dosage of Hydroxychloroquine and Chloroquine to Convert Symptomatic RT-PCR Positive Severe Acute Respiratory Syndrome Coronavirus 2 Coronavirus Infectious Disease 2019 Patients to RT- PCR-Negative as a Means to Reduce Hospitalization Rate	April 15, 2020	April 30, 2020	No	Mayo Hospital Lahore, Pakistan	Yes	Quadruple	Yes	400	100	**800**	**400**	**5**	mg	**–**
4,303,507	Chloroquine/ Hydroxychloroquine Prevention of Coronavirus Disease (COVID-19) in the Healthcare Setting	April 2020	April 2021	No	University of Oxford, UK	Yes	Double	Yes	40,000	20,000	**600**	**200**	**90**	mg	**–**
4,354,870	ff Label Study to Evaluate the Efficacy of HCQ for Pre-exposure Prophylaxis (PrEP) to Prevent Severe Acute Respiratory Syndrome Coronavirus 2 (SARS-CoV-2) Infection Among Health Care Workers (HCWs) Who Are at High Risk of Occupational Exposure to SARS-CoV-2	April 3, 2020	August 1, 2020	Yes	NYU Langone Health New York, New York, USA	No	None	No	350	300	**600**	**200**	**90**	mg	**–**
4,330,495	Randomized, Controlled, Double-blind Clinical Trial Comparing the Efficacy and Safety of Chemoprophylaxis With Hydroxychloroquine in Patients Under Biological Treatment and/or JAK Inhibitors in the Prevention of SARS-CoV-2 Infection	April 6, 2020	Nov 6, 2020	No	Instituto de Investigación Marqués de Valdecilla, Spain	Yes	Double	Yes	800	400	**400**	**400**	**180**	mg	**–**
4,352,946	Protecting Health Care Workers From COVID-19 With Hydroxychloroquine Pre-exposure Prophylaxis: A Randomized, Placebo-controlled Trial	April 24	June 24, 2020	No	GeoSentinel Foundation	Yes	Quadruple	Yes	374	187	**400**	**400**	**60**	mg	**–**
4,328,285	Chemoprophylaxis of SARS-CoV-2 Infection (COVID-19) in Exposed Healthcare Workers: A Randomized Double-blind Placebo-controlled Clinical Trial	April 14, 2020	Nov 30, 2020	Yes	CHU de Saint-Etienne Institut Pasteur, France	Yes	Triple	Yes	1200	300	**400**	**400**	**60**	mg	**–**
4,344,379	Prevention of SARS-CoV-2 in Hospital Workers s Exposed to the Virus	April 15, 2020	July 31, 2020	Yes	Assistance Publique - Hôpitaux de Paris, France	Yes	Double	Yes	900	300	**400**	**400**	**40**	mg	**–**
4,331,834	Pre-Exposure Prophylaxis With Hydroxychloroquine for High-Risk Healthcare Workers During the COVID-19 Pandemic: A Unicentric, Double-Blinded Randomized Controlled Trial	April 3, 2020	Oct 3, 2020	Yes	Barcelona Institute for Global Health, Spain	Yes	Quadruple	Yes	440	220	**400**	**400**	**4**	**400**	**24**
4,328,961	Efficacy of Hydroxychloroquine for Post-exposure Prophylaxis (PEP) to Prevent Severe Acute Respiratory Syndrome Coronavirus 2 (SARS-CoV-2) Infection Among Adults Exposed to Coronavirus Disease (COVID-19): a Blinded, Randomized Study	March 2020	Sept 30, 2020	Yes	University of Washington New York University, USA	Yes	Double	Ascorbic acid	2000	1000	**400 D1-D3**	**200**	**14**	mg	**–**
4,341,441	Will Hydroxychloroquine Impede or Prevent COVID-19: WHIP COVID-19 Study	April 7, 2020	June 30, 2020	Yes	Detroit, Michigan, USA	Yes	Triple	Yes	3000	1500	**400**	**200**	**56**	**Or 6.5** **mg/kg/week**	**8**
4,336,748	Low-dose Hydroxychloroquine for Primary Prophylaxis Against SARS-CoV-2 in Health-care Workers - a Randomized, Double-blind, Controlled Trial	April 2020	July 2020	No	Medical University of Vienna, Austria	Yes	Triple	Yes	440	220	**200**	**200**	**28**	mg	**–**
4,318,015	Chemoprophylaxis With Hydroxychloroquine in Healthcare Personnel in Contact With COVID-19 Patients: A Randomized Controlled Trial	April 14, 2020	Dec 31, 2020	Yes	National Institute of Respiratory Diseases, Mexico	Yes	Quadruple	Yes	400	200	**200**	**200**	**60**	mg	**–**
4,334,928	Prevention of SARS-CoV-2 (COVID-19) Through Pre-Exposure Prophylaxis With Tenofovir Disoproxil Fumarate/Emtricitabine and Hydroxychloroquine in Healthcare Personnel: Randomized Clinical Trial Controlled With Placebo	April 15, 2020	June 30, 2020	No	Hospital Universitario Ramón y Cajal, Madrid, Spain,	Yes	Double	Yes	4000	1000	**200**	**200**	**84**	mg	**–**
4,349,228	Assessment of the Efficacy and Safety of (HCQ) as a Prophylaxis for COVID19 for Health Professionals	April15, 2020	July 15, 2020	No	Hôpital Abderrahmane Mami-Ariana Tunis, Tunisia	Yes	Single	Yes	530	265	**200**	**200**	**60**	mg	**–**
4,328,467	Pre-exposure Prophylaxis for SARS-Coronavirus-2: A Pragmatic Randomized Clinical Trial	April6, 2020	August 2020	Yes	Minneapolis, Minnesota, USA	Yes	Quadruple	Yes	3500	1166	**800**	**–**	**1**	**400**	**12**
4,328,467	Pre-exposure Prophylaxis for SARS-Coronavirus-2: A Pragmatic Randomized Clinical Trial	April6, 2020	August 2020	Yes	Minneapolis, Minnesota, United States, USA	Yes	Quadruple	Yes	3500	1166	**800**	**–**	**1**	**800**	**12**
4,333,225	A Prospective Clinical Study of Hydroxychloroquine in the Prevention of SARS- CoV-2 (COVID-19) Infection in Healthcare Workers After High-risk Exposures	April 3, 2020	July 30, 2020	invitation	Baylor University Medical Center Dallas, Texas, USA	No	None	No	360	360	**800**	**–**	**1**	**400**	**7**
4,347,889	Prophylactic Hydroxychloroquine vs Vitamin C in Healthcare Workers at Risk of COVID-19: A RCT	April 20	Dec 30, 2020	No	Stony Brook University, New York USA	Yes	Single	Vitamin C	1212	606	**800**	**–**	**1**	**400**	**12**
4,352,933	ChemoPROphyLaxIs For covId-19 Infectious Disease (the PROLIFIC Trial)	April 2020	Oct 31, 2020	No	Cambridge University Hospitals NHS Foundation Trust, UK	Yes	Quadruple	Yes		330	**800 D1 & D2**	**200**	**90**	**or 400/wk after loading**	**12**
4,341,441	Will Hydroxychloroquine Impede or Prevent COVID-19: WHIP COVID-19 Study	April 7, 2020	June 30, 2020	Yes	Detroit, Michigan, USA	Yes	Triple	Yes	3000	1500	**400**	**200**	**56**	**Or 6.5** **mg/kg/week**	**8**
4,345,653	Hydroxychloroquine as Chemoprevention for COVID-19 for High Risk Healthcare Workers	April 14, 2020	April 8, 2021	Yes	Hackensack Meridian Health - JFK Medical Center Edison, New Jersey, USA	No	None	No	45	45	**400**	**–**	**1**	**400**	**3**

Among the included studies, all but three were randomized and parallel and most of them (74%, 23/31) were double-blinded to quadruple-blinded studies. On the 27th of April, 55% (17/31) of them were recruiting. Estimated enrolment in HCQ arm was from 45 to 20.000 participants, with a median of 380 participants and a total of 45.728 persons receiving HCQ.

Regarding HCQ regimen, 61% (19/31) of the included studies used an HCQ loading dose, followed by daily (14/19) or weekly (5/19) doses. The range of the loading doses was from 400 to 1400 mg on day 1. The most common daily doses were 400 mg (12/31 (39%)) and 200 mg (9/31 (29%)); a 600 mg daily dose was less common and was recorded for only 13% (4/31) of the studies. The remaining six studies used weekly doses of 400 mg. Regarding the duration of prophylaxis, it was highly variable, ranging from 5 to 180 days (median = 40 days) for daily regimen, and 3 to 24 weeks for weekly regimen (median = 12 weeks). Of note, the most frequent prophylactic regimen (6/31 (19%)) was an HCQ loading dose of 800 mg on day 1, followed by HCQ 400 mg for four additional days. Among the studies (*n* = 5) that did not use a loading dose but a 400 mg daily dose, duration of prophylaxis was highly diverse from 4 to 180 days (median = 60). For the studies (*n* = 2) that reported a 200 mg daily dose, one study used a loading dose of 800 mg on day 1 and 2, followed by 90 days of 200 mg HCQ, and the other one used a loading dose of 400 mg from day 1 to 3, followed by 14 days of 200 mg HCQ.

## 4 Discussion

More than 40 randomized clinical trials have been registered in less than 2 months from 13 different countries to answer the same question: should we used HCQ to protect health-care workers from the COVID-19 consequences? This very active recording in ClinicalTrials.gov demonstrates the huge interest of the scientific community regarding this question. Indeed, the debate continues to rage regarding the use of HCQ for COVID-19 and we need to shed more light based on clinical evidence. At the present time, the debate is still a non-documented speculation that will be ended in the next few months.

The positive point regarding the high diversity of HCQ regimen among recorded clinical studies is that nearly all the possible regimens are under evaluation. The negative point of the high diversity in HCQ dosage and duration of prophylaxis could be that the conclusion of these different studies may be conflicting. Indeed, it would be surprising that a 200 mg daily dose during one month would have the same efficacy and the same ratio benefit/risk than a 600 mg daily dose during three months. As a consequence, the final analysis of these trials should be done through an extensive reading of the results in regards to the clinical design, rather than quickly glancing a 140 characters-based social media message announcing the failure or success of a drug against a disease.

The authors declare no conflict of interest.

## References

[1] Picot S, Marty A, Bienvenu A-L, Blumberg LH, Dupouy-Camet J, Carnevale P, et al. Coalition: Advocacy for prospective clinical trials to test the post-exposure potential of hydroxychloroquine against COVID-19. One Health. 4 avr 2020;100131. 10.1016/j.onehlt.2020.100131 PMC7128742 32292817

[2] COVID-19 Clinical Research Coalition. Electronic address: nick.white@covid19crc.org. Global coalition to accelerate COVID-19 clinical research in resource-limited settings. Lancet. 25 avr 2020;395(10233):1322–5. 10.1016/S0140-6736(20)30798-4 PMC7270833 32247324

[3] Pagliano P, Piazza O, De Caro F, Ascione T, Filippelli A. Is Hydroxychloroquine a possible post-exposure prophylaxis drug to limit the transmission to health care workers exposed to COVID19? Clin Infect Dis. 24 mars 2020. 10.1093/cid/ciaa320 PMC7184439 32211764

[4] NIH ClinicalTrials.gov 41 Studies for Hydroxychloroquine prophylaxis | COVID, Prevention, Severe Acute Respiratory Syndrome Coronavirus 2 SARS-CoV-2 https://clinicaltrials.gov/ct2/results?cond=COVID&term=hydroxychloroquine+prophylaxis&cntry=&state=&city=&dist=.

